# Fracture toughness of different monolithic zirconia upon post-sintering processes

**DOI:** 10.4317/jced.58717

**Published:** 2021-10-01

**Authors:** Niwut Juntavee, Apa Juntavee, Thipradi Phattharasophachai

**Affiliations:** 1 Department of Prosthodontics, Faculty of Dentistry, Khon Kaen University, Khon Kaen, Thailand; 2 Division of Pediatric Dentistry, Department of Preventive Dentistry, Faculty of Dentistry, Khon Kaen University, Khon Kaen, Thailand; 3 Division of Biomaterials and Prosthodontics Research, Faculty of Dentistry, Khon Kaen University, Khon Kaen, Thailand

## Abstract

**Background:**

Surface treatments are expected to be a reason for alteration in fracture resistance of zirconia. This study evaluated the effect of post-sintering processes on the fracture toughness of different types of monolithic zirconia.

**Material and Methods:**

Classical- (Cz) and high-translucent (Hz) monolithic zirconia discs (1.2 mm thickness, 14 mm in Ø) were prepared, and randomly divided for surface treatments with 1) as-glazed (AG); 2) finished and polished (FP); 3) finished, polished, and overglazed (FPOG); and 4) finished, polished, and heat-treated (FPHT) technique (n=15/group). Fracture toughness (KIC) was determined with indentation fracture toughness method at load 1 kg for AG, FPOG and 10 kg for FP, FPHT with 15 sec dwelling time. Weibull analysis was applied for survival probability, Weibull modulus (m), and characteristic toughness (K0). Microstructures were examined with SEM and XRD. ANOVA and multiple comparisons were determined for significant differences (α=0.05).

**Results:**

The mean±sd value of KIC (MPa.m1/2), m, and K0 were 1.60±0.19, 7.27, 1.71 for CzAG; 9.57±0.89, 9.97, 10.96 for CzFP; 1.61±0.15, 10.56, 1.68 for CzFPOG; 6.45±0.31, 20.31, 6.60 for CzFPHT; 1.45±0.13, 10.91, 1.51 for HzAG; 6.58±0.24, 27.00, 6.70 for HzFP; 1.24±0.05, 23.90, 1.27 for HzFPOG; and 5.07±0.16, 30.51, 5.15 for HzFPHT. The KIC was significantly affected by the post-sintering process, type of zirconia (*p*<0.05). The Cz indicated a significantly higher KIC than Hz. The FP significantly enhanced KIC, while OG was unable to raise KIC. HT reduced KIC due to reverse phase transformation.

**Conclusions:**

Post-sintering processes caused alteration in fracture resistance of zirconia. Fracture toughness was enhanced with FP, but not with either OG or HT process for both Cz and Hz. Surface treatment of zirconia through a finished-polished process is recommended, while glazing and heat-treated are not suggested.

** Key words:**Fracture toughness, glazing, heat treatment, polishing, post-sintering process, zirconia.

## Introduction

All-ceramic restoration has become popular and plays a significant role in contemporary restorative dentistry as a result of modern technology of computer-assisted design and computer-assisted manufacturing (CAD-CAM) and the advancement of ceramic nanomaterials, that are capable of providing aesthetic and load-bearing restorations (1,2). Zirconia has been introduced as a substructure for fixed dental prostheses owing to its strength, biocompatibility, and natural inert white crystalline oxide of zirconium (3). It consists of three crystalline phases: monoclinic (m), tetragonal (t), and cubic (c). The m-phase is stable at room temperature up to 1170◦C, turns to t-phase beyond 1170ºC, and changes to c-phase at 2370ºC. The t-phase is a stronger crystalline structure than the m-phase (4). Therefore, it is necessary to stabilize the t-phase at room temperature by adding some chemical stabilizing oxides such as 3%mol yttrium-oxide (Y2O3) particles, leading to a 3-yttrium partially stabilized tetragonal zirconia polycrystal (3Y-TZP). When the zirconia is subjected to stress-initiated cracks, high compressive stress can be induced at the crack tips, leading to t- → m-phases transformation with 4-4.5% volumetric expansion, resulting in a crack inhibition phenomenon, known as transformation toughening (2,4). The stress can be generated from the heat upon surface grinding, which eventually induces superficial surface alterations, crack, crack propagation, premature aging, as well as phase transformation (5). Although zirconia has been developed for being a white color, the primitive zirconia is quite opaque, it needs to be veneered with porcelain to achieve a natural-looking tooth appearance. However, porcelain chipping and delamination is the most common complication of porcelain veneered zirconia. The evolutions of zirconia tend to be monolithic zirconia, more translucent, and life-like characteristics (6). The classical translucence monolithic 3Y-TZP (Cz) has been developed to eliminate the opaqueness of traditional zirconia. Currently, the monolithic zirconia is introduced as it is an intrinsic pre-colored shade to match the tooth color after sintering. The restoration can be fabricated with the reduced amount of tooth preparation to be as little as 0.5–0.7 mm thickness (3). The translucency of zirconia can be achieved by increasing the sintering temperature, reduction of alumina, or increasing Y2O3 at a higher concentration. Adding 5%mol of Y2O3 to zirconia yields a high amount of c-phase with a smaller grain size of 5-yttrium partially stabilized zirconia (5Y-PSZ) (7,8). The 5Y-PSZ showed better translucency and also aging resistance than the classical and translucent 3Y-TZP (8,9). However, the 5Y-PSZ was decreased in strength and possesses less t-phase, thus less capable of stress-induced phase transformation toughening (9). The phase transformation majorly occurs in the form of t- → m-phase, which enhances the strength of zirconia. The transformation toughening rarely occurs in the situation of reduced t-phase. Hence, the strength and fracture toughness of translucent zirconia was reduced. It was reported that the 5Y-PSZ possessed only half the strength of 3Y-TZP (7,8). However, the aging of material still is less effect on the high-translucent zirconia (Hz) (8,9).

Post-sintering processes are clinical procedures that every clinician needs to perform on the zirconia restoration before delivery to the patient. The restorations need to be ground, adjusted, finished, polished, glazed, or heat-treated (10-13). The strength of Hz seems to reduce after receiving the surface modification due to containing a lower t-phase. The phase transformation of Hz can occur in form of either t- or c- to rhombohedral (r-) phase. The r- or distorted t-phase was found in zirconia after receiving surface modification for example sandblasting, machining, ground with a diamond bur, and polished procedure (10,11). The presence of r-phase was originally found from the x-ray diffraction (XRD) pattern as the asymmetric or left-hump broad peak at a diffraction angle (2Ɵ degree) 30º (12). It was observed at all levels of grinding and for various amounts of yttria dopant, even in the fully stabilized c-zirconia (13). However, the r-phase appearance was disappeared by heat treatment at 1000°C for one hour (10), which indicated the possibility of reverse transformation of the r-phase. The relationship of the r-phase on strength has never been reported. However, the volume change of approximately 3.9% for t- → r-phase and 5.2% for c- →r-phase transformation was indicated (10).

Fracture toughness is a mechanical characteristic of brittle material to resist crack propagation under applied stress. It is measured by the amount of energy required for fracture which is quantified by the stress-intensity factor (KIC) (4,14). Fracture toughness can be accessed by an indentation fracture (IF) method that can be performed by inducing the load to create surface fracture using 136º diamond pyramid Vicker’s indenter (15-19). The crack lengths are in an inverse proportion to the toughness of the material (15,18). The indentation load was different for each material, so, the optimum load should verify and beforehand (16,17). Since the post-sintering processes of monolithic zirconia are unavoidable procedures. There are few studies about the effect of the post-sintering processes on fracture toughness of zirconia (14). There is no study till now related to fracture toughness of Hz. There is no standard protocol for monolithic zirconia adjustment after sintering. The controversy exists regarding the effects of clinical adjustment by grinding with burs, finishing, polishing, glazing, or heat treatment on fracture resistance. Hence, the purpose of this study was to investigate fracture toughness of Cz and Hz after receiving different post-sintering processes. The null hypothesis was that glazing, finishing and polishing, overglazing after polishing, and heat treatment after polishing of either classical or high translucent monolithic zirconia would not affect differences in fracture toughness.

## Material and Methods

-Preparation of zirconia specimens

The zirconia blanks were milled into a cylindrical shape with an 18 mm diameter (Ø) from pre-colored classical translucent monolithic zirconia (Cz, BruxZir™ Shaded, Prismatik Dental craft, Irvine, CA), and high translucent monolithic zirconia (Hz, BruxZir™ Anterior shaded) and sectioned into a circular disc shape of 1.6 mm in thickness using a low-speed diamond cutting machine (Isomet®1000, Beuhler, Lake Buff, IL). The zirconia discs were compensated for sintering shrinkage with the enlargement factor of 1.2302 for Cz and 1.2334 for HZ. The discs were ground flat to remove surface irregularities with silicon carbide abrasive grit # 500, 800, and 1200, respectively, with water coolant, then sintered in a furnace (inFire HTC, Sirona, Bensheim, Germany) according to the manufacturer’s firing schedules. One hundred and twenty zirconia discs of thickness 1.2±0.2 mm and Ø 14±0.2 mm were derived. The discs were sandblasted with 50 µm alumina oxide abrasive with a pressure of 30 psi at a distance of 15 mm from the blasting tip and then cleaned in distilled water. The mixture of glazing paste and liquid (IPS e.max® Ceram, Ivoclar Vivadent, Schaan, Liechtenstein) was applied over the blasted surface and fired in a ceramic furnace (Programat P-310, Ivoclar Vivadent) to produce a glazed surface.

-Post-sintering surface treatment

**Figure 1:**
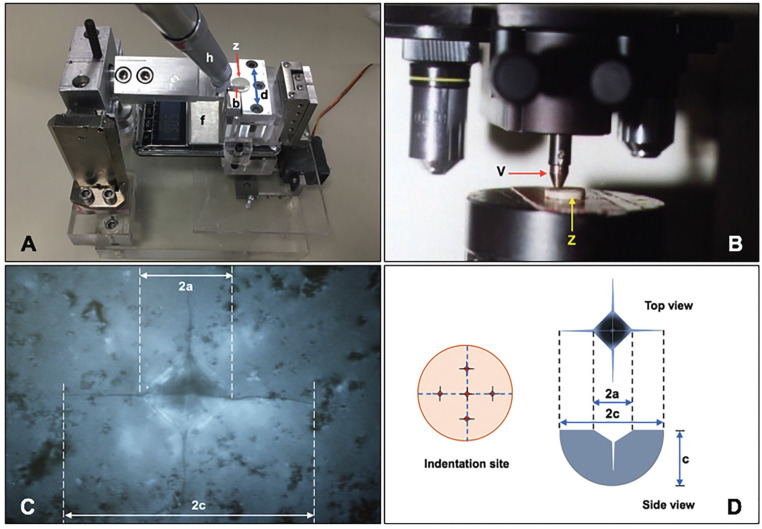
Custom-made machine (A) was used for controlling force (f) and direction (d) during finishing and polishing on the surface of zirconia (z) with bur (b) in the fixture mounted hand-piece (h). Fracture toughness was determined using Vicker’s hardness machine (B) by placing the zirconia disc (z) against the Vicker’s indenter (v) to create crack (C). Five indentations were performed at equidistance between the indentation point and the center of each disc, and the crack length was measured and used to calculate fracture toughness (D).

All specimens were randomly divided into four groups according to post-sintering surface treatment: AG (as-glazed), FP (finished and polished), FPOG (finished, polished, and overglazed), and FPHT (finished, polished, and heat-treated) groups. The specimens in the AG group did not receive any surface treatment. The specimens in the FP group were ground by fine diamond finishing bur (882F, Frank Dental, Gmund, Germany) by an air-rotor with a speed of 400,000 rpm and water coolant 50 ml/min. The contact pressure was exactly 50 grams, and the finishing time for each step was 30 seconds in a continuous stroke. The horizontal movement was conducted in one direction with the custom-made load and direction-controlled machine with a fixture for holding the grinding handpiece (Fig. 1A). The bur was changed to a new one for every single specimen. Then, the specimens were finished with a vitrified bonded diamond abrasive bur (Dura green® HP0155, Shofu, Kyoto, Japan) with the speed of a straight handpiece at 20,000 rpm, in continuous strokes and sweeping motions. The polishing procedure was performed by the three-step diamond-impregnated silicone polishing system: coarse, medium, and fine grit (ZilMaster®, Shofu), to produce a polished surface. The specimens in the FPOG group were ground, finished, and polished similar to those in the FP group and then ultrasonic cleaned, steam cleaned, and finally overglazed, as previously described. The specimens for the FPHT group were finished, polished, and cleaned similarly to the FP group, and heat-treated at 910°C for one minute in a porcelain furnace.

Determination of fracture toughness

Indentation fracture (IF) was performed in the Vicker’s hardness tester (Zwick, Stourbridge, UK) by placing the treated surface of zirconia against the indenter (Fig. 1B) and loaded with 10 kgs for Cz groups and 1Kg for Hz groups, with 15 seconds dwelling time to create crack (Fig. 1C). Five indentations were performed at equidistance between the indentation point and the center of each disc. The crack length was measured [Figure 1(D)] by using the optical microscope (Olympus, Tokyo, Japan), and used to calculate fracture toughness (KIC) by equations 1 and 2 (20).

K_IC=0.016 (E⁄Hυ)1/2 (P⁄c3/2) )............. Equation 1

Hυ=P/2a2................... Equation 2

Where: KIC is the fracture toughness (MPa•m1/2), E is Young’s modulus (GPa), Hυ is hardness (MPa), a is indent half-diagonal (m), *P* is indentation load in newton, c is the radius of crack length (m), 

-Statistical analysis

The mean and standard deviation (sd) of KIC for each group of monolithic zirconia were calculated, compared, and then further analyzed using ANOVA in conjunction with a post hoc Bonferroni multiple comparisons using statistical software (SPSS version 22, Chicago, IL) to determine significant differences in the KIC of monolithic zirconia materials with different post-sintering processes. The result was considered statistically significant at a 95% confidence interval (CI). Weibull analysis was used to determine the reliability of KIC and to estimate characteristic toughness (K0) as well as the Weibull modulus (m) using Weibull++® statistics (ReliaSoft, Tucson, AZ) according to equation 3.

Pf(KIC)=1-exp (-KIC/K0 )m…………….....Equation 3

where: Pf is toughness probability, KIC is fracture toughness, K0 is characteristic toughness, and m is Weibull modulus.

-Microscopic examination

The specimens were coated with gold at a current of 10 mA and a vacuum of 130 m-torr for three minutes, then dried in a desiccator, and finally evaluated the microstructures with a scanning electron microscope (SEM, Hitachi S-300N, Osaka, Japan). The crystalline phases of zirconia were determined by using an X-ray diffractometer (XRD, D8 Advance-Bruker, Ettlinger, Germany). The crystal structures were examined at a diffraction angle of 20–90º with a 0.02º step size per second intervals using copper k-alpha radiation. The phase was analyzed by cross-reference with the standards database and determined the intensity of peaks using X’Pert Plus software (Philips, Almelo, Netherlands).

## Results

**Figure 2:**
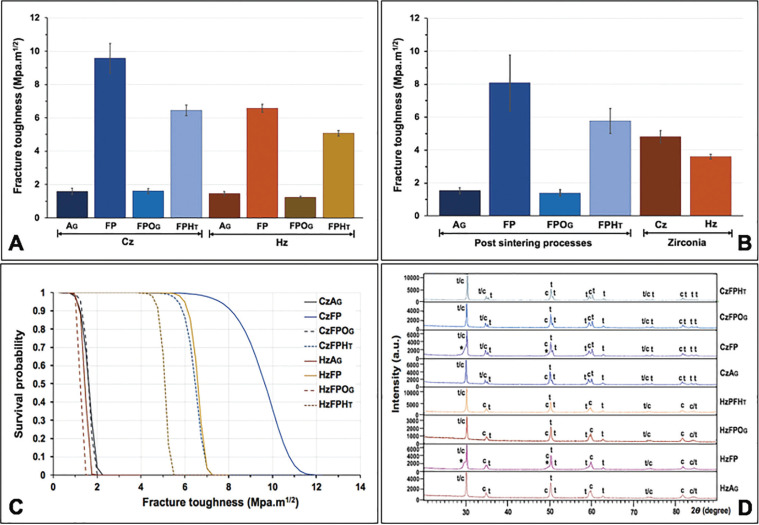
Fracture toughness (A,B), Weibull survival probability (C), and X-ray diffraction pattern (D) of the classical (Cz) and high translucent zirconia (Hz) upon post-processing surface treatment with as-glazed (AG), finished and polished (FP), finished, polished and overglazed (FPOG) and finished, polished and heat-treated (FPHT) techniques. The star (*) indicated a left-hump broad peak of r-phase or distorted t-phase.

The mean, sd, 95% CI of KIC, K0, and m for each group are shown in Table 1 and Fig. 2A. ANOVA indicated a statistically significant difference in KIC of zirconia upon post-sintering processes, type of zirconia, and their interaction (*p*<0.05) (Table 2). The results indicated that the Cz groups possessed significantly higher KIC than the Hz group (*p*<0.05) (Fig. 2B). The post-sintering processes revealed a statistically significant effect on the KIC of monolithic zirconia (*p*<0.05). The mean±sd values of KIC of zirconia upon post-sintering surface treatment with AG, FP, FPOG, and FPHT were 1.53±0.18, 8.07±1.70, 1.39±0.21, and 5.76±0.77 MPa•m1/2, respectively (Fig. 2B). Post-hoc multiple comparisons showed significant differences in KIC among post-sintering processes, except for AG and FPOG (Table 3). No significant difference in KIC was observed among groups of glazed surface for both Cz and Hz (CzAG, CzFPOG, HzAG, and HzFPOG) (Table 3). However, a significant difference in KIC among groups of non-glazed surface for both Cz and Hz zirconia (CzFP, CzFPHT, HzFP, and HzFPHT) (Table 3). For non-glaze surfaces, the CzFP group revealed the highest KIC, while the HzFPHT group showed the lowest KIC. However, both CzFPHT and HzFP groups had no significant difference in KIC. In addition, all groups with the non-glazed surface was a significantly higher KIC than other groups with glazed-surface. The post-sintering process with FP and FPHT significantly enabled KIC enhancement for both Cz and Hz but did not affect other processes. Weibull analysis of the reliability of KIC for both Cz and Hz upon different post-sintering processes indicated the “m” varied among groups and indicated their relative survival probability of the material upon fracture toughness (Table 1, Fig. 2C).

**Table 1:**
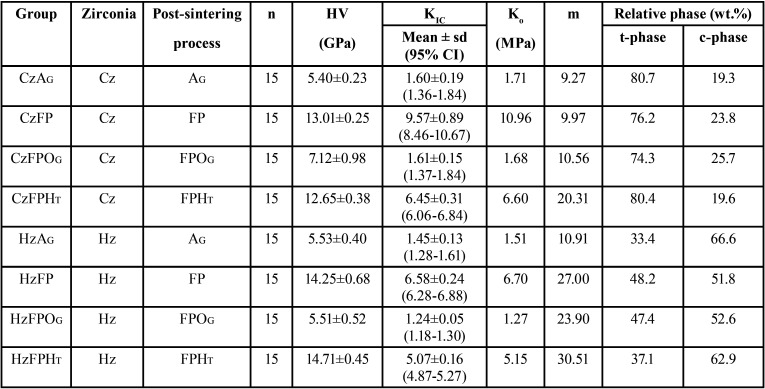
Mean, standard deviation (sd) of fracture toughness (KIC), 95% confidence interval (CI), Vicker hardness (HV), characteristic fracture toughness (Ko), Weibull modulus (m), and relative tetragonal (t-) and cubic (c-) phase content (wt.%) of the classical (Cz) and high translucent zirconia (Hz) upon post-processing surface treatment with as-glazed (AG), finished and polished (FP), finished, polished and overglazed (FPOG) and finished, polished and heat-treated (FPHT) techniques.

**Table 2:**
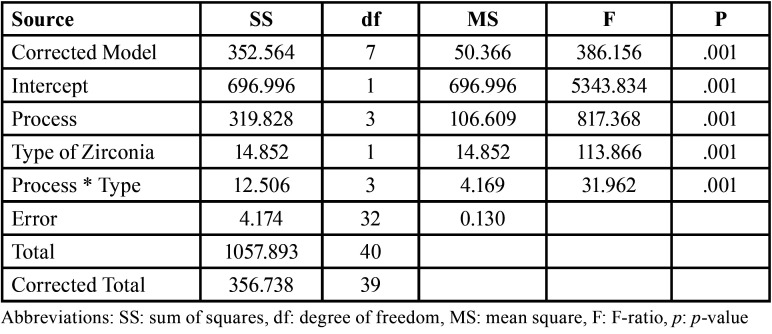
An analysis of variance (ANOVA) of fracture toughness of the different types of zirconias upon different post-sintering processes.

**Table 3:**
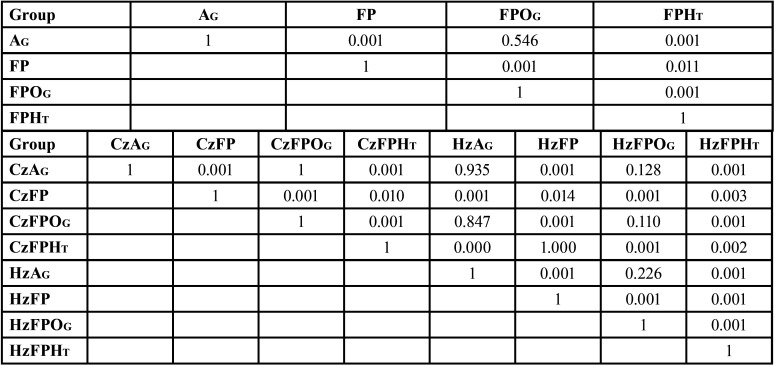
Multiple comparisons of fracture toughness of conventional translucent (Cz) and high translucent (Hz) monolithic zirconia after treated surface through different post-processing surface treatment with as-glazed (AG), finished and polished (FP), finished, polished, and overglazed (FPOG), and finished, polished, and heat-treated (FPHT) techniques.

**Figure 3:**
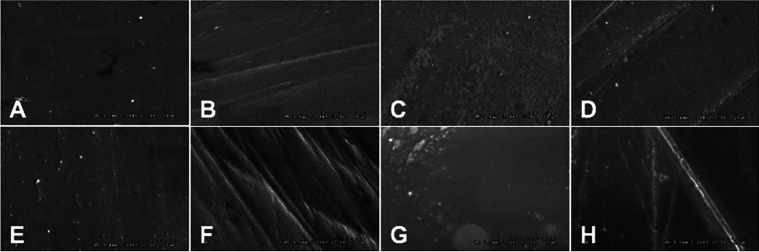
SEM photomicrographs of topographic surfaces (A-H) (3.0Kx) of the classical (A-D) and high translucence zirconia (E-H) upon post-processing treated surface with as-glazed (A,E), finished and polished (B,F), finished, polished and overglazed (C,G) and finished, polished and heat-treated (D,H) techniques.

The XRD analysis of the crystalline contents and phases of the Cz and Hz was illustrated in Table 1 and Figure 2D. Both Cz and Hz zirconia demonstrated a large amount of t- and c-phase, with no m-phase existed. The dominant peaks of the t-phase were observed at the 2θ degree of 30.2°, 34.8°, 35.34°, 50.19°, and 59.54° that correlated with the 101-, 002-, 110-, 111-, and 103-crystalline planes, respectively. The dominant peaks of the c-phase were detected at the 2θ degree of 29.9°, 34.68°, 49.5°, and 59.54° which corresponded to the 111-, 020-, 022-, and 131- crystalline planes, respectively. It was observed for the broad peaks of t-phase at 101- plane for both CzFP and HzFP groups which referred to r- or distorted t-phase of zirconia (*). The XRD patterns of Cz mostly indicated the t- phase and a minor amount of the c- phase, vis versa for Hz. The relative phase concentration was shown in Table 1. The SEM photomicrographs revealed the irregularity of the surfaces in the CzAG, CzFPOG, HzAG, and HzFPOG group, due to small particles of glazing material, which possibly indicated the incomplete adhesion of glazing materials and seems to have a void inside the glazed layer (Fig. 3A,C,E,G). The topography of the CzFP, CzFPHT, HzFP, and HzFPHT groups consisted of scratch lines in one direction, without a distinguished difference (Fig. 3B,D,F,H). Meanwhile, the FPOG group (Fig. 3C,G) exhibited a smooth surface rather than a polished surface or FH groups (Fig. 3B,F).

## Discussion

This study indicated that post-sintering processes significantly affected fracture toughness of different types of monolithic zirconia. Therefore, the null hypothesis was rejected for the post-sintering processes, types of zirconia, and their interactions. Fracture toughness is the characterizing value of stress absorption in the material at the crack site before the catastrophic failure occurs (14,16). The IF method is a simple and non-destructive technique that frequently uses when the material possesses high hardness or high strength (19). The selection of proper load is crucial to prevent forming crack branches and chipping of the material surface (17,19).

Post sintering adjustment of restoration through grinding, finishing, and polishing procedures is a stepwise method, which is necessary to proceed from the coarsest grit to the finest grit size. These procedures are needed to achieve a smooth, mirror-like surface that provides less susceptibility to bacterial plaque accumulation and minimizes deleterious effects of low-temperature degradation (LTD) and wears of antagonist natural dentition (21,23-26). Such surface adjustment is unavoidable even if the restoration is close to perfect after milling and sintering. The restoration must be adjusted clinically during the trial process, before cementation (22,23). The ground zirconia showed significant deterioration in its long-term, which is negatively affected by aging (5). Some studies claim that grinding by coarse diamond burs improves the strength because of the transformation toughening mechanism (25,26). Some studies have found no significant correlation between roughness and strength, especially when using a small diamond grit size (5,23,25). This study used fine grit diamond grit size of 38-45 µm, combined with a proper polishing procedure and coolant may not influence the t- → m-phase transformation because it probably causes a smaller rise in surface temperature while treating the zirconia surface (26). However, the XRD pattern of the FP-zirconia had the left hump broad shoulder peaks of r-phase at 2Ɵ degree of 30° and 50° for both Cz and Hz zirconia, indicating the possibility of phase transformation of t- or c-phase to r-phase, as seen in other studies (10,26). Both t- → r-phase and c- → r-phase transformations caused a volume increasing approximately 3.9% and 5.2%, respectively, which are capable of inducing a compressive stress layer within the 20 µm for both Cz and Hz zirconia (10,11). However, the r-phase was eradicated after heat treatment in this study, as supported by other studies (10,11). This indicates that the occurrence of the r-phase leads to a fracture toughness enhancement for FP groups. The sequential multistep polishing procedures are recommended and widely used because of their ability to produce high-gloss surfaces in zirconia comparable to glazed surfaces. The gloss finishing was also produced by applying glaze material, but the fracture toughness results were significantly lowered, possibly because of moisture in the glazing mixture and heat from the glaze firing (23). It was found that the mixture of glazing components trapped air bubbles within the glazed layer. The air bubbles inside the glazed layer may represent a trigger point of failure. Moreover, the glass matrix in the mixed glazing paste did not properly melt or adhere to the zirconia, as it does with glass-based ceramics. Nevertheless, in areas demanding high esthetics, glazing shall be applied to the zirconia because the polishing procedure can decrease its brightness and produce disharmonious color compared to the natural teeth (26). Heat treatment can reverse the phase transformation when heat is applied at 910°C for one minute. In this study, the increase of the t-phase was found upon the heat-treated process, compared to FP and FTOG. The heat treatment process seems to be less affected with Hz, probably because of the lower ability of the Hz to change phase. This result was consistent with that of other studies (20,22). The SEM showed the surface irregularities of the FPHT which did not differ from those of the FP, which means that the heat treatment did not repair the flaws or porosity of the surface. Thus, the LTD or aging of zirconia can occur and may weaken the restoration in the long term. Aging may be reduced by heat treatment, which is helpful for the long-term service, as found in another study (11).

Two major types of the surface were exhibited upon post-sintering processes; the glazed surface (AG and FPOG groups) and polished surface (FP and FPHT groups). The glazed surface consisted of low-fusing fluorapatite glass-ceramics that cannot withstand the load as much as the zirconia surface does. The 1 kg load on the glazed surface was capable of producing crack length, which similar to the glass-based dental ceramics (19). While 10 kgs load was needed for the polished surface to produce crack length, but quite shorter than the glazed surface. Interestingly, the finishing and polishing procedure (FP groups) showed the higher fracture toughness, while the glazed zirconia showed lower fracture toughness (FPOG and AG groups) for both Cz and Hz. Moreover, there is some area around the indented area of the glazed surface that could be the delamination of the glazed layer from the zirconia underneath. This appearance is probably related to the improper adherence between the glazed layer and zirconia (1,27,28). Traini *et al*. 2014 showed that fracture toughness of polished zirconia with fine polishing silicone bur was lower than the coarse polishing silicone bur and machining zirconia. However, the surface roughness from machining and coarse polishing zirconia was not acceptable in a clinical situation (29). The glazed surface indicated lower KIC which meant that the glazed surface possessed lower fracture resistibility. When the glazed surface was cracked, the exposure site can pass the fluid to the zirconia, which made underneath zirconia susceptible to LTD as supported by another study (30).

Conventional translucence zirconia has high KIC especially in the CzFP group and lowers respectively for CzFPHT, CzAG, and CzFPOG groups. This related with the phase transformation as evidenced by the broad peaks of r-phase at 101-crystalline plane for CzFP, which responsible for the t- → r- phase transformation mechanism, resulting in KIC increasing, compared to the CzAG group. The r-phase might affect the crack healing ability or the ability to resist crack propagation (10,11). The CzFPHT group indicated lower KIC than the CzFP, indicating that the heat treatment at 910 °C for one minute affected the KIC of a polished surface. This is probably related to the partially reverse phase transformation mechanism from r- → t- phase for the CzFPHT group, in which the CzFPHT group has only a sharp peak of t-phase at 30°, whereas no broad peak of r-phase as the CzFP zirconia does. However, the KIC for the CzFPHT group was still higher than that of the CzAG and CzFPOG groups. Similar evidence was shown with Hz, in which the highest KIC was found for the HzFP group and lowered respectively for HzFPHT, HzAG, and HzFPOG groups. There is no significant difference in KIC between the HzAG and HzFPOG. The HzFP group has the left-hump shoulder broad peak at 30°, 50°, and 60° area, which refers as r-phase. Thus, the KIC enhancement for the HzFP group resulted from the t- → r-phase transformation mechanism. The r-phase was disappeared as the Hz was a heat-treated surface. This probably indicated that there is partially reverse phase transformation from the r- → t-phase in case that the polished surface was further heat-treated. However, the KIC for the HzFPHT group was still higher than that of the HzAG and HzFPOG group. A study indicated significantly lower KIC of 5Y- PSZ (4.8±0.58 MPa•m1/2) than 3Y-TZP (6.9±0.98 MPa•m1/2) [9]. However, the 5Y-PSZ has higher translucency significantly and lower ability phase transformation as evaluated from aging behavior [9]. Hence, the hydrothermal stability of 5Y-PSZ was better than the 3Y-TZP. This situation would lead to different transformation toughening abilities because of the different phase composition in each type of zirconia. The Hz zirconia contains more c-phase and the phase changing ability was lower (10,11). Although the c-phase could change to r-phase, the transformation-induced volumetric change was lower than the transformation of t- → m-phase or t- → r-phase (10). As a result, the heat-treated procedure showed significantly lower KIC than the finished-polished procedure.

The Weibull analysis provided the m, K0, and survival probability. The m in ceramic was used to determine the reliability of the material and the distribution of flaws. A higher m had higher reliability or homogenous distribution of flaws (29). Most of the lower m values in this study were found in the glazed surface groups, which may cause by the glazed material. The K0 was different in each post-sintering process. The FP had the highest K0, which means that a polished surface can be better fracture surface resistance. Moreover, when comparing the K0 of the OG and AG groups, both treatments were found to be comparable. It showed that the group subjected to grinding and then glazing exhibited lower K0 due to the incompatibility between the glazed layer and the zirconia itself (22). Hence, the glazing procedure is not necessary. This can reduce the treatment period and obviate any complex procedures. The glazing shall be done in a high-esthetic-demanding area to provide natural-seeming color to the adjacent teeth. Nevertheless, the restoration should be checked during the trial process to ensure no defect exists before cementation.

## Conclusions

Within the limitations of this study, the fracture toughness was influenced by types of zirconia, post-sintering processes. Each post-sintering process resulted in a different superficial zirconia surface. The bare zirconia that occurred from the multi-stepwise finishing and polishing process provided the highest fracture toughness due to the phase transformation ability and reduction of surface flaws. The overglazing provides a luster surface, but it does not enhance fracture toughness because it is based on low-fusing nano-fluorapatite glass ceramics that is indicated for glazing on the zirconia. Moreover, the adherence between glazing and zirconia was occurred by mechanical retention, so, this layer can chip out and removed over time. The heat treatment after polishing provides the bare zirconia surface, so, the fracture toughness is high. The heat treatment influences the reverse phase transformation, then the fracture toughness was lower than the finished-polished process. Nevertheless, the long-term service life of heat-treated zirconia may better than only the grinding and polishing process. Monolithic zirconia which can be used in the posterior (conventional zirconia) and anterior region (high-translucent zirconia), the finishing polishing procedure is unavoidable, it provides adequate strength to deliver to the patient while the overglazing or heat treatment is an optional procedure.
